# Mechanical phenotype of cancer cells: cell softening and loss of stiffness sensing

**DOI:** 10.18632/oncotarget.4173

**Published:** 2015-05-19

**Authors:** Hsi-Hui Lin, Hsiu-Kuan Lin, I-Hsuan Lin, Yu-Wei Chiou, Horn-Wei Chen, Ching-Yi Liu, Hans I-Chen Harn, Wen-Tai Chiu, Yang-Kao Wang, Meng-Ru Shen, Ming-Jer Tang

**Affiliations:** ^1^ Department of Physiology, National Cheng Kung University, Tainan, Taiwan; ^2^ Institute of Basic Medical Sciences, National Cheng Kung University, Tainan, Taiwan; ^3^ Department of Biomedical Engineering, National Cheng Kung University, Tainan, Taiwan; ^4^ Department of Cell Biology and Anatomy, National Cheng Kung University, Tainan, Taiwan; ^5^ Department of Pharmacology, National Cheng Kung University, Tainan, Taiwan

**Keywords:** matrix stiffness, mechanical phenotype, stiffness sensing, cell stiffness, caveolin-1

## Abstract

The stiffness sensing ability is required to respond to the stiffness of the matrix. Here we determined whether normal cells and cancer cells display distinct mechanical phenotypes. Cancer cells were softer than their normal counterparts, regardless of the type of cancer (breast, bladder, cervix, pancreas, or Ha-Ras^V12^-transformed cells). When cultured on matrices of varying stiffness, low stiffness decreased proliferation in normal cells, while cancer cells and transformed cells lost this response. Thus, cancer cells undergo a change in their mechanical phenotype that includes cell softening and loss of stiffness sensing. Caveolin-1, which is suppressed in many tumor cells and in oncogene-transformed cells, regulates the mechanical phenotype. Caveolin-1-upregulated RhoA activity and ^Y397^FAK phosphorylation directed actin cap formation, which was positively correlated with cell elasticity and stiffness sensing in fibroblasts. Ha-Ras^V12^-induced transformation and changes in the mechanical phenotypes were reversed by re-expression of caveolin-1 and mimicked by the suppression of caveolin-1 in normal fibroblasts. This is the first study to describe this novel role for caveolin-1, linking mechanical phenotype to cell transformation. Furthermore, mechanical characteristics may serve as biomarkers for cell transformation.

## INTRODUCTION

Our previous study showed that epithelial cells cultured on a flexible collagen gel exhibited a more contracted cell morphology and were able to actively pull collagen fibers, which subsequently resulted in extensive apoptosis [1, 2]. Stiffening the collagen gel through glutaraldehyde-mediated cross-linking facilitated cell extension on the gel and attenuated the collagen gel-induced apoptosis. These data imply that the physical properties of the collagen gel regulate apoptosis. In contrast, cancer cells were resistant to collagen gel-induced apoptosis. Interestingly, Yu-Li Wang and his colleagues also demonstrated that nontransformed cells cultured on a flexible matrix showed a decrease in proliferation and an increase in apoptosis [3]. Overexpression of Ha-Ras increased cell survival on a flexible matrix [3]. Therefore, we hypothesized that cancer cells might exhibit different biomechanical characteristics from normal epithelial cells, allowing them to maintain their growth regardless of the matrix stiffness.

Matrix stiffness has a large impact, similar to chemical stimuli, on the regulation of cell behaviors such as survival, proliferation, differentiation, and migration [4]. A pioneering study by Engler et al. showed that mesenchymal stem cells (MSCs) change their shape and progress toward lineage-specific differentiation in response to culture on matrices that present different, physiologically relevant matrix stiffnesses [5]. To behave differently on matrices of varying stiffness, cells must have the ability to detect and respond to the mechanical resistivity of the extracellular environment; this ability is known as “stiffness sensing” [4]. Our previous study showed that cells detect and respond to the matrix stiffness through the dynamic regulation of integrin clustering, focal adhesion complex formation, and actin filament remodeling [6]. Furthermore, the tension and integrity of the actin filaments play important roles in both mechanoresponses and mechanotransduction [4, 6, 7]. Notably, cell transformation is characterized by alterations in cellular morphology and migration ability, mainly due to changes in the distribution of cytoskeleton filaments and adhesion proteins [8]. These findings prompted us to investigate whether cancer cells are defective in their “stiffness sensing” ability, enabling their survival and proliferation regardless of the matrix stiffness.

Caveolin-1 (Cav1), a 21-24-kDa protein involved in caveolae formation, is downregulated in many tumor-derived or oncogene-transformed cells [9]. Mechanistically, Cav1 has been reported to inhibit cell proliferation by impeding a variety of proteins that are associated with cell proliferation and survival, including Src, epidermal growth factor receptor, protein kinase C, and endothelial nitric oxide synthase [9]. Suppression of Cav1 is sufficient to induce cellular transformation, and re-expression of Cav1 results in the reversal of this phenotype in fibroblasts. In addition to its role as a tumor suppressor, Cav1 has also been linked to the regulation of focal adhesions and integrin-mediated actin remodeling. Both of these processes were widely studied with regard to mechanotransduction [10-12]. Therefore, we were interested in whether the reduction of Cav1 contributes to the change of cell mechanics in cancer cells.

In the present study, we attempted to clarify whether cancer cells display different mechanical phenotypes, particularly with regard to cell stiffness and stiffness sensing. Cancer cells and Ha-Ras^V12^-transformed cells were softer than their normal counterparts and exhibited a loss of stiffness sensing, characterized by a failure to adjust their stiffness in response to that of the matrix and growth in a matrix-stiffness-independent manner. Cell elasticity and stiffness sensing ability were positively correlated with the expression level of Cav1. Cav1-upregulated RhoA activity and ^Y397^FAK phosphorylation direct actin cap formation, which subsequently contributes to both mechanosensation and mechanotransduction in fibroblasts. The results of this study demonstrate a novel role for Cav1, linking the mechanical phenotype to the control of cell transformation. Furthermore, the biomechanical characteristics of cells, i.e. cell softening and the loss of stiffness sensing, serve as important biomarkers of a cancer cell “phenotype”.

## RESULTS

### Cancer cells/tissues are softer than their normal counterparts

**Figure 1:**
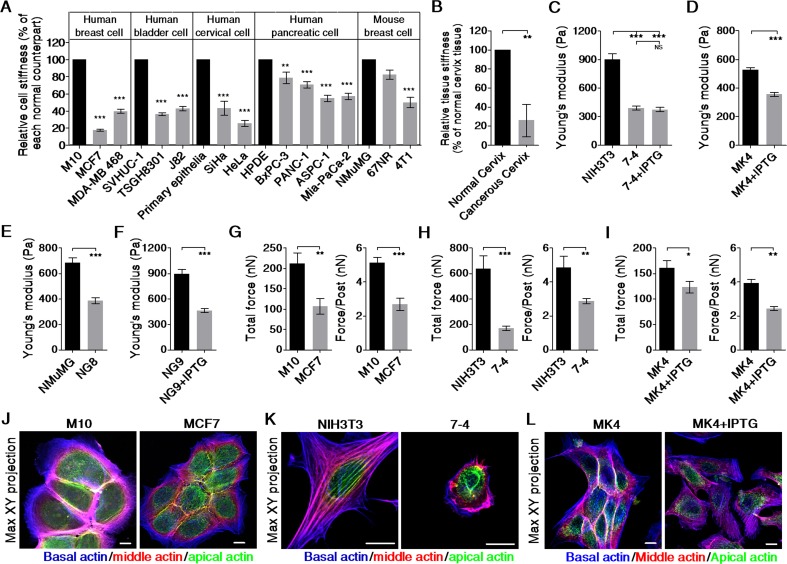
Cancer cells are softer and display lower traction force than their normal counterparts Cells were cultured on a collagen-coated glass coverslip overnight. The effective Young's moduli of cells were measured by Bio-AFM. (**A**) Cancer cell lines (gray bar) were softer than their normal counterparts (black bar). The results were normalized to corresponding reference values, which are for their normal counterpart (black bar). Data are presented as mean relative value ± SEM. (**B**) Cancerous cervix tissues are softer than the adjacent normal cervix tissues. The results were normalized to corresponding reference values, which are for adjacent normal tissue (black bar). Data are presented as mean relative value ± SEM. Ha-Ras^V12^ overexpression resulting in cell softening was demonstrated in (**C**) NIH3T3 and 7-4 cells, (**D**) MK4 cells (derived from MDCK epithelia), (**E** and **F**) NG8 and NG9 (both derived from NMuMG epithelia). (**G**) MCF7 cells exhibited lower traction than M10 cells did. Ha-Ras^V12^ overexpression in 7-4 cells (**H**) and in MK4 cells (**I**) led to a decrease in traction force. Data are expressed as mean ± SEM. The representative Max XY projection images for (**J**) M10 and MCF7 cells, (**K**) NIH3T3 and 7-4 cells, and (**L**) MK4 cells with or without IPTG stained with phalloidin. Actin fibers in the apical, middle, and basal regions of the cell were recolored green, red, and blue, respectively. *P < 0.05, ** *P* < 0.01, *** *P* < 0.001, NS = not significant. Scale bar = 10 μm.

To test whether normal cells and cancer cells exhibit different physical properties, we used Bio-AFM to probe the biomechanical characteristics of the cells. Primary normal epithelial cells and well-characterized normal and cancer cell lines derived from human breast, bladder, cervix, and pancreas tissues and from mouse breast tissue were evaluated. Figure 1A shows that the cancer cell lines (gray bar) were significantly softer than their normal counterparts (black bar). Moreover, the majority of the cervical cancer tissues were significantly softer than their adjacent normal tissues (Figure 1B). To determine whether cancer cell softening is directly related to oncogene activation, we compared the elastic moduli of cells with or without the IPTG-induced Ha-Ras^V12^. The 7-4 cell line, which is derived from NIH3T3 fibroblasts, contains Ha-Ras^V12^ due to leakage. These cells displayed significant increases in proliferation, migration, and anchorage-independent growth (Supplementary Figure 1A–1D) compared with the parental NIH3T3 cells. Additionally, as expected, 7-4 cells were much softer than the parental cell line (Figure 1C). However, induction of Ha-Ras^V12^ by IPTG elicited an increase in ERK activation but did not induce cell softening and transformation. In MK4 cells, IPTG treatment induced Ha-Ras^V12^ expression and ERK activation, which then resulted in cell transformation, as confirmed by the cells’ resistance to anoikis, as well as their anchorage-independent growth and migration, invasion, proliferation, and foci formation abilities (Supplementary Figure 2A–2G). The MK4 cells became significantly softening 20–24 h after IPTG administration (Figure 1D). Notably, this persistent decrease in the elastic modulus preceded the acquisition of the transformation phenotype and was reversed after withdrawing IPTG (Supplementary Figure 2H). Constitutive and IPTG-inducible overexpression of Ha-Ras^V12^ in NG8 and NG9 cells, respectively (Supplementary Figure 3A–3B), resulted in cell softening at 24 h (Figure 1E and 1F) followed by transformation, as confirmed by the anchorage-independent growth and foci formation capacity of the cells (Supplementary Figure 3C and 3D). Furthermore, K-Ras^V12^ overexpression in normal human pancreatic ductal cells also resulted in cell softening and transformation (data not shown). When cultured on a fibronectin-coated microfabricated post-array-detector (mPAD), cancer cells or Ha-Ras^V12^-transformed cells (gray bar) deflected the micropost to a lesser extent than their normal counterparts (black bar), which resulted in a lower total force and a lower traction force for each post (Figure 1G–1I). In summary, a correlation between cancer cells or transformed cells and cell softening was demonstrated by AFM indentation and traction force analysis in both fibroblasts and epithelial cells.

Cell stiffness is highly correlated with the cellular traction force and the cell spreading area, and these characteristics are regulated by actin polymerization and myosin II-mediated cytoskeleton contractility [13, 14]. To show the entire architecture of the actin filaments, we generated the Max XY projection images from a stack of recolored confocal images of cells with actin filament staining. M10 cells displayed intense marginal actin bundles and junctional actin belts, which were colocalized with the continuous adherens junction belt. MCF7 cells displayed weak, thin marginal actin bundles and discontinuous junctional actin belts (Figure 1J). In fibroblasts, the NIH3T3 cells presented a flattened phenotype and had the most robust structures, including parallel thick actin bundles (actin cap) wrapping over the nucleus. In contrast, the 7-4 cells showed a rounded phenotype with prominent actin-rich membrane protrusions in lieu of an actin cap (Figure 1K). Upon IPTG administration, MK4 cells dramatically changed within 20 h from typical epithelial colonies with marginal actin bundles and junctional actin belts to scattered and motile single cells with prominent basal stress fibers (Figure 1L). In summary, our data show that cancer or transformed cells are softer than their normal counterparts, which might be correlated with disturbed or disorganized actin filaments.

### Cancer cells/Ha-Ras^V12^-transformed cells did not respond to variations in matrix stiffness

**Figure 2:**
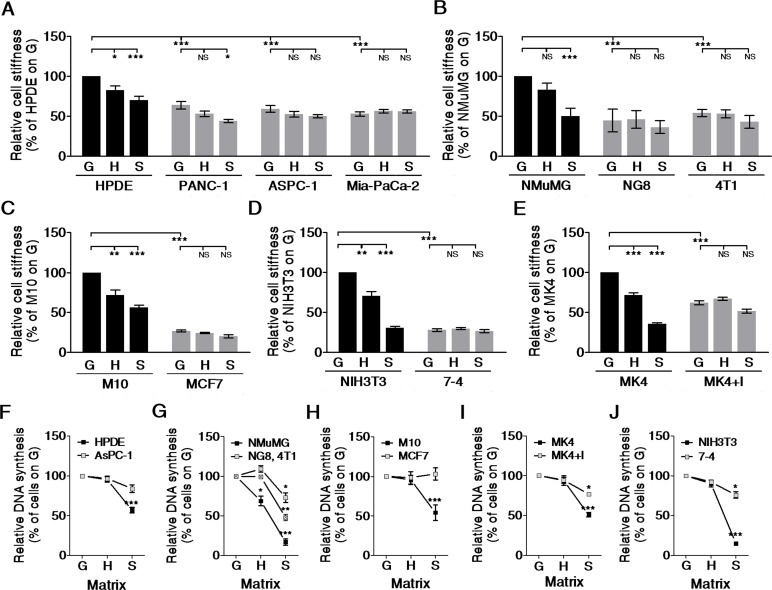
Cancer/Ha-Ras^V12^-transformed cells did not alter cell stiffness on matrices of varying stiffness and were insensitive to soft matrix-induced growth arrest Cells were plated on type I collagen-coated matrices, including a glass dish (G, E>G kPa), hard PA gel (H, E=20 kPa), and soft PA gel (S, E=0.2 kPa) overnight. The effective Young's moduli of cells were measured by Bio-AFM. The data were normalized to corresponding reference values, which were for their normal counterparts (black bar) cultured on G for 24 h. Data are presented as mean relative value ± SEM. The relative cell stiffness was compared in (**A**) pancreatic normal (HPDE) and cancer (PANC-1, ASPC-1, and Mia-PaCa-2) epithelial cells, (**B**) mouse mammary gland normal (NMuMG), Ha-Ras^V12^ overexpressed (NG8) and cancer (4T1) epithelial cells, (**C**) human mammary gland normal (M10) and cancer (MCF7) epithelial cells, (**D**) mouse normal (NIH3T3) and Ha-Ras^V12^ overexpressed (7-4) fibroblasts, and (**E**) canine renal normal (MK4) and Ha-Ras^V12^ overexpressed (MK4+I) epithelial cells. For cell proliferation, the DNA synthesis was quantified by the ratios of EdU-positive cells to Hoechst 33342-positive cells, as described in the Materials and Methods. The results were normalized to corresponding reference values, which are for cells (black square) cultured on G. Data are presented as mean relative value ± SEM. The relative cell proliferations were compared in (**F**) HPDE and ASPC-1 cells, (**G**) NMuMG, NG8, and 4T1 cells, (**H**) M10 and MCF7 cells, (**I**) NIH3T3 and 7-4 cells, and (**J**) MK4 and MK4+IPTG. *P < 0.05, ** *P* < 0.01, *** *P* < 0.001, NS = not significant.

Cell shape and stiffness are strongly influenced by matrix stiffness [15-18]. To evaluate the effect of matrix stiffness on cell stiffness, cells were cultured on matrices of varying stiffness, and their stiffness was measured by Bio-AFM indentation. Normal cells (black bar) exhibited significantly altered stiffness in response to the stiffness of the matrix they were cultured on, while cancer cells or Ha-Ras^V12^-transformed cells did not (Figure 2A–2E). These results demonstrate that cancer cells were not only softer than normal cells, but they were also insensitive to variations in matrix stiffness. To investigate the effect of matrix stiffness on cell growth, we evaluated cell proliferation based on DNA synthesis, which was monitored by Click-iT^®^EdU incorporation. In normal cells (black squares), the DNA synthesis rate was highest in cells cultured on a glass dish and declined with decreasing matrix stiffness (Figure 2F–2J). In general, normal cells displayed a marked growth arrest on soft gel. By contrast, the DNA synthesis rate of cancer cells or transformed cells (gray squares) exhibited a low dependence on the stiffness of the matrix. Collectively, these results confirm the physiological importance of a soft matrix for arresting normal cell growth. In addition, cancer cells or transformed cells were insensitive to soft matrix-induced growth arrest.

### Cav1 level determines the formation of the actin cap, which is relevant to cell elasticity and stiffness sensing in fibroblasts

**Figure 3:**
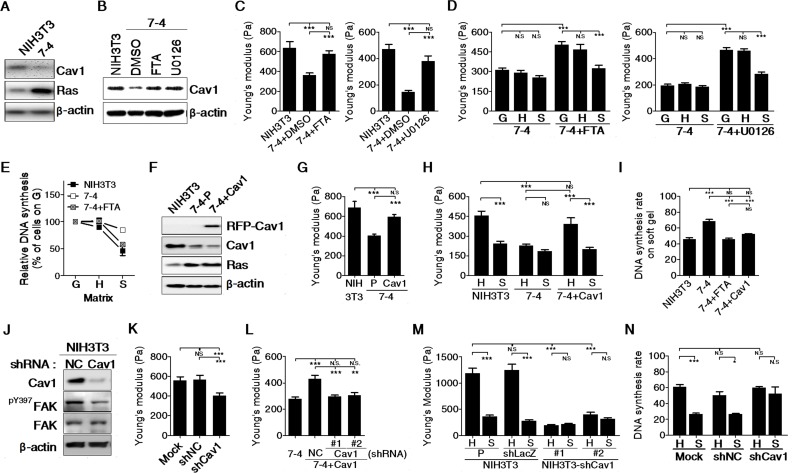
The levels of Cav1 is positively correlated with cell stiffness and stiffness sensing ability in NIH3T3 cells (**A**) Ha-Ras^V12^ overexpressed 7-4 cells displayed decrease Cav-1 in protein level. (**B**) Cav1 expression was restored in 7-4 cells by treatment with Ras inhibitor farnesylthiosalicylic acid (FTA) and MEK1/2 inhibitor (U0126). Both FTA and U0126 treatment abrogated Ha-Ras^V12^-induced (**C**) cell softening, (**D**) inability to change cell stiffness on matrices of varying stiffness, (**E**) escape from soft matrix-induced growth arrest. (**F**) Representative immunoblots for Cav1 in NIH3T3, 7-4 and 7-4+Cav1 cells. In 7-4+Cav1 cells, Cav1 overexpression abolished Ha-Ras^V12^-induced (**G**) cell softening, (H) inability to change cell stiffness on matrices of varying stiffness, (**I**) escape from soft matrix-induced growth arrest. (**J**) Representative immunoblots for Cav1, ^pY397^FAK, and FAK expression in NIH3T3 cells stably transfected with negative control (NC) shRNA or Cav1 shRNA. β-actin served as loading control. Cav1 knockdown in NIH3T3 cells and 7-4+Cav1 cells resulted in (K and **L**) cell softening, (**M**) inability to change cell stiffness on matrices of varying stiffness, (**N**) escape from soft matrix-induced growth arrest. All data were expressed as mean ± SEM. *P < 0.05, ** *P* < 0.01, *** *P* < 0.001, NS = not significant.

To sense the stiffness of the matrix, cells must first mechanically probe it. Cell-matrix adhesions are thus critical for the regulation of stiffness sensing. We found that Cav1, a key component involved in β1 integrin-dependent mechanotransduction, was suppressed in most cancer cell lines (Supplementary Figure 4). Induction of Ha-Ras^V12^ also suppressed Cav1 in 7-4 cells and MK4 cells (Figure 3A). We then evaluated whether Cav1 was associated with changes in cell mechanics, including cell elasticity and stiffness sensing, in Ha-Ras^V12^-transformed cells. In 7-4 cells, both a Ras inhibitor (FTA) and a MEK1/2 inhibitor (U0126) restored both Cav1 expression and cell stiffness (Figure 3B and 3C). FTA- and U0126-treated 7-4 cells regained stiffness sensing ability to alter cell stiffness significantly in response to the stiffness of the matrix. Additionally, these cells displayed growth arrest on a soft gel (Figure 3D and 3E). Both inhibitors also suppressed Ha-Ras^V12^-induced transformed phenotype (Supplementary Figure 5A–5C). To further confirm the role of Cav1 in the regulation of the mechanical properties of cells, 7-4 cells were transfected with RFP-Cav1 (Figure 3F). 7-4+Cav1 cells not only became stiffening, they also regained stiffness sensing ability to alter cell stiffness significantly in response to the stiffness of the matrix and exhibited growth inhibition on a soft gel (Figure 3G–3I). In addition, cell migration and anchorage-independent growth were suppressed in 7-4+Cav1 cells (Supplementary Figure 5D and 5E). On the other hand, knockdown of Cav1 by shRNA caused cell softening in NIH3T3 and 7-4+Cav1 cells (Figure 3J–3L). When cultured on matrices of varying stiffness, NIH3T3/shCav1 cells did not alter their stiffness and proliferate in a stiffness-independent manner (Figure 3M and 3N). The impairment of Cav1 function by methyl-β cyclodextrin (MβCD), the caveolae disruptor, also led to cell softening in NIH3T3 cells (data not shown). As expected, NIH3T3/shCav1 cells displayed increases in cell migration, invasion, and anchorage-independent growth (Supplementary Figure 5F–5H). These results indicate that Cav1 was positively correlated with the mechanical properties of cells, including cell stiffness and stiffness sensing.

**Figure 4:**
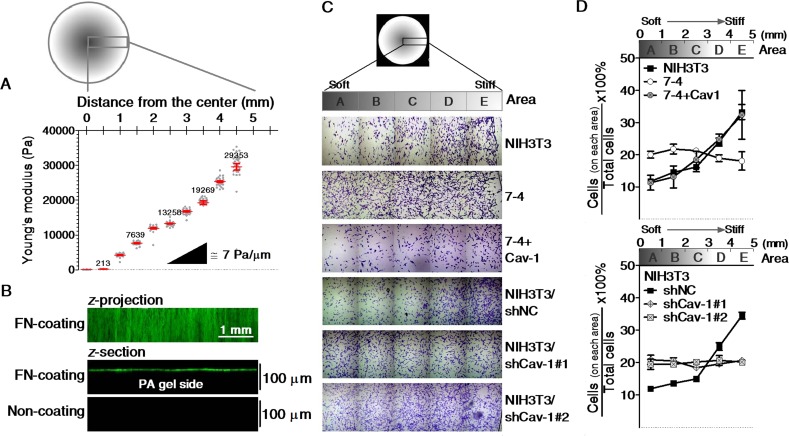
The spatial distributions of cells cultured on stiffness gradient PA gels A representative spatial map of the distance from the center of the gradient PA gel versus (**A**) the elasticity (Pa) characterized by AFM, (**B**) the distribution of fibronectin of the gradient gel functionalized with (top and middle) or without (bottom) fibronectin (green) as demonstrated by the confocal Z-projection and Z-sectional images. (**C**) Mosaic crystal violet staining images of cells on gradient PA gels for three days. (**D**) The number of cells on each indicated area were counted and normalized to the total number of cells on the whole gradient PA gel. Scale bar = 1 mm.

**Figure 5:**
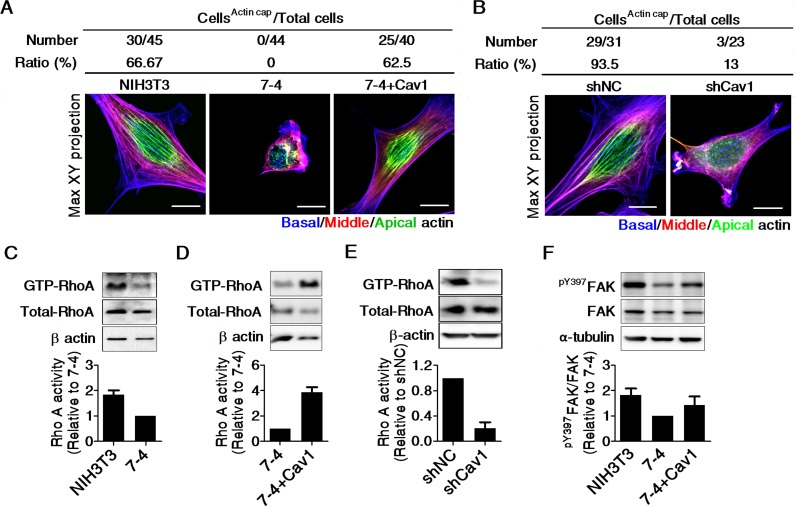
The levels of Cav1 is positively correlated with Rho A activity, the phosphorylation of ^Y397^FAK, and actin cap formation in NIH3T3 cells The quantification of cells with actin cap formation and the representative Max XY projection images of (**A**) NIH3T3, 7-4, and 7-4+Cav1 cells, and (**B**) NIH3T3/shNC and NIH3T3/shCav1. Cells were plated on type I collagen-coated dishes overnight and then fixed and stained with Alexa 594-phalloidin. Actin fibers in the apical, middle, and basal regions of the cell were recolored green, red, and blue, respectively. Representative RhoA immunoblots and relative RhoA activity in (**C** and **D**) NIH3T3, 7-4 and 7-4+Cav1 cells, and (**E**) NIH3T3/shNC and NIH3T3/shCav1. Cells were plated for 20 h and extracted for Rho activity assay, as described in Materials and Methods. The decrease in RhoA activity in the 7-4 cells was reversed by Cav1 overexpression. (**F**) Representative immunoblots for ^pY397^FAK, FAK, and α-tubulin and the quantification results in NIH3T3, 7-4 and 7-4+Cav1 cells. All data are expressed as relative mean ± SEM from two or three independent experiments. Scale bar =10 μm.

To confirm the role of Cav1 in the regulation of stiffness sensing, we cultured cells on an FN-coated gradient PA gel. Figure 4C shows the spatial distribution of crystal violet-stained cells on FN-coated gradient PA gels after three days of culture. The cell distribution is expressed as the number of cells in the indicated region relative to the total number of cells on the gradient PA gel. The quantification results show that NIH3T3, NIH3T3/shNC, and 7-4+Cav1 cells preferentially accumulated in stiffer regions of the PA gel, while 7-4 cells and two clones of NIH3T3/shCav1 cells were evenly distributed on the gradient PA gel, regardless of the matrix stiffness (Figure 4D). The actin cap links the extracellular milieu to the nucleus and provides a fast and effective mechanotransduction system in mesenchymal cells [19]. In 7-4 cells, the actin cap completely disappeared but was restored by the re-expression of Cav1 (Figure 5A). Notably, the expression levels of Cav1 were positively correlated with RhoA activity (Figure 5C and 5D) and ^Y397^FAK phosphorylation (Figure 5F), both of which are required for actin cap formation. In NIH3T3 cells, the actin cap was destroyed by shRNA knockdown of Cav1 (Figure 5B), which was accompanied by the suppression of ^Y397^FAK phosphorylation and RhoA activity (Figure 3J and 5E). In summary, our results demonstrate that Cav1 promotes the formation of actin caps by elevating the phosphorylation of ^Y397^FAK and RhoA activity in fibroblasts. In particular, our findings highlight the importance of Cav1 in the regulation of cell mechanics.

## DISCUSSION

Cells’ ability to undergo transformation is accompanied by various molecular changes leading to alterations in the organization of the cytoskeleton. Therefore, the observation of cell stiffness might enable allow for the effective detection and evaluation of cancer cells. In this paper, we demonstrated that cancer cells are softer than their normal counterparts by AFM indentation and traction force analysis. Similar results have been shown previously [20-22]. However, these studies compared cells derived from different genetic or pathological backgrounds. To circumvent this problem, we used an IPTG-inducible gene expression system to confirm that the induction of Ha-Ras^V12^ elicited a morphological change and that cell softening occurs prior to cell transformation. Although we were able to demonstrate a lower Young's modulus in cancer cells, further studies are needed to measure cancer cells directly within cancer tissues. Cancer tissue is particularly complex with large changes in cell phenotype, extracellular matrix (ECM) composition, and chemical molecules. In general, tumor tissues tend to be stiff, which is a result of accumulating ECM and increasing tension [23]. In this paper, we demonstrate that cancerous cervix tissues are softer than their adjacent normal cervix tissue *in situ*, which is consistent with the finding that cancer cells are softer than normal cells *in vitro*. Cervical cancer often occurs on the superficial layer of the cervix without much ECM deposition [24, 25]. Therefore, we can probe live cancer and normal cells directly from specimens of cervical tissue and the adjacent normal tissues without destroying the tissue integrity to show the real cell stiffness *in situ*.

“Stiffness sensing” renders cells susceptible to their mechanical environment [4]. Matrix-stiffness-dependent changes are correlated with cellular behaviors, such as survival, proliferation, migration, and differentiation [2, 4]. Our previous study showed that normal cells spread much more extensively on a stiff matrix than on a soft matrix [6] by altering their cytoskeletal organization and focal adhesions. These differences may reflect the changes in cell stiffness (Figure 2). When cultured on a stiffness-gradient PA gel, the normal cells tended to accumulate in stiffer areas (Figure 4), probably due to durotaxis [26] and soft matrix-induced growth arrest [27]. In contrast, cancer cells or Ha-Ras^V12^-transformed cells failed to tune their stiffness to comply with that of the matrix they adhered to. Furthermore, their proliferation and migration were matrix-stiffness-independent (Figure 2 and 4). Taken together, these data suggest that “stiffness sensing” is lost or defective in cancer cells and Ha-Ras^V12^-transformed cells. Physiological tissue stiffness was considered a general cell cycle inhibitor, and comparable increases in tissue stiffness facilitated at sites of cell proliferation *in vivo* and *in vitro* [27]. The maintenance of tissue stiffness is thus fundamental for the physiological function of the organs. Our results provide the innovative insight that the loss of stiffness sensing allows transformed cells to evade the inhibition of cell growth induced by natural physical barriers. Overall, changes in biochemical molecules and biomechanics should be considered together to improve our understanding of the unregulated growth of transformed cells and the initiation of tumorigenesis. The loss of stiffness sensing could also explain why cancer cells escape from soft matrix-induced apoptosis [2, 3].

Although the stiffness optima for different kinds of normal cells vary widely, it is generally true that cell spread and proliferation increase with the stiffness of the matrix. Contrarily, past studies on the response of cancer cells to variation in matrix stiffness have a diverse set of results. Using PDMS with tunable stiffness and topography, Tzvetkova-Chevolleau et al. showed that the morphology and migration of transformed SaI/N fibroblastic cells appeared insensitive to variations in matrix stiffness [28]. The separate study demonstrated that cancerous prostate and melanoma cells spread out and proliferate better on soft PDMS than on stiff PDMS [29]. Feng et al. showed that the sensitivity of MCF7 cells to the cytotoxicity of cisplatin and Taxol was more effective on rigid glass/PDMS than on soft PDMS [30]. Tilghman et al. analyzed the “growth profile” of several cancer cell lines on PA gel of varying rigidity and grouped them into “rigidity independent” (cells growth equally on both soft and stiff matrices) and “rigidity dependent’’ (cells growth increases with increasing matrix stiffness) [31]. They suggested that the “rigidity profile” is an intrinsic property of each cancer cell line. Kostic et al. demonstrate a differential rigidity response in the single-cell populations (SCPs) derived from a highly invasive MDA-MB 231 cell line [32]. They found bone-targeting SCPs displayed preferential growth and invasiveness on rigid matrix, while lung-targeting SCPs preferred to proliferate and be invasive on soft matrix and nonmetastatic SCPs proliferated regardless of matrix stiffness. The results revealed that the matrix stiffness response in various SCPs correlates with the tissue tropism displayed *in vivo*. Thus, the differential matrix rigidity responses from different studies might be due to the diverse intrinsic property of the cancer cell lines used.

The disruption of tissue stiffness through extracellular matrix deposition or crosslinking interferes with tissue development and disease progression [33]. Stiffening the ECM enhanced β1 integrin expression and potentiated transforming growth factor (TGF)-β-dependent ECM crosslinking or deposition, which accelerated EMT progression and the development of malignant phenotypes [23, 34-36]. TGFβ-induced epithelial-mesenchymal transition (EMT) has been shown to promote the cell motility, invasiveness, and metastasis of cancer cells [37]. We found that TGFβ-primed transformed cells increased their stiffness and regained their ability to sense and respond to the stiffness of the matrix (Supplementary Figure 6). TGFβ-elevated β1 integrin and α-smooth muscle actin (α-SMA) have been reported to function almost exclusively in actin filaments to mediate the generation of traction forces and mechanotransduction [6, 38]. Thus, the mechanical phenotype of cancer cells might be changed by altered TGFβ expression at a late stage of the malignancy process to regain stiffness sensing and change their growth pattern from “stiffness-independent growth” to “stiffness-dependent growth”.

The actin cap functions as a physical link between the nucleus and mature focal adhesion sites to maintain the nuclear homeostatic balance and the internal functions of chromatin structure [39]. The actin cap is present in a wide range of adherent cells and is disrupted in several human diseases, including laminopathies and cancer [19]. In this study, we show that Cav1 is involved in RhoA activation and ^Y397^FAK phosphorylation, which are both required for actin cap formation in fibroblasts. Mih et al. showed that inhibition of actomyosin contractility selectively promotes cell proliferation on soft matrices [40]. In particular, inhibition of actomyosin contractility suppressed actin cap formation and focal adhesion maturation [19, 41]. We also found that inhibition of actomyosin contractility resulted in cell softening and the loss of stiffness sensing (data not shown). Taken together, our results confirm that Cav1 is an important factor involved in regulating the mechanical phenotypes of cells. In addition to regulating focal adhesions and integrin-mediated actin remodeling, Cav1 was reported to target the actin-binding protein filamin, a candidate for mediating the cellular responses to matrix stiffness [42]. Thus, it is reasonable that the reduction of the level of Cav1, by either oncogenic activation or shRNA, caused cell softening and the loss of stiffness sensing in normal fibroblasts, and re-expression of Cav1 restored the mechanical properties of Ha-Ras^V12^-transformed fibroblasts.

Nevertheless, approximately 90% of human cancers occur in epithelial tissues. Epithelial cells show a distinct polarity due to the well-formed cell junctions. Cell junctions connect to actin filaments and play a critical role in regulating cortical tension and maintaining the mechanical coupling between cells, both of which contribute to tissue morphogenesis and homeostasis [43, 44]. In the early stage of cancer, cell junctions are often disrupted [45]. In normal epithelia, Cav1 has been demonstrated to recruit the E-cadherin/β-catenin complex to the membrane and stabilize cell-cell adhesion [46, 47]. Previous studies showed that the re-expression of Cav1 restored the epithelial phenotype by suppressing the EMT signaling pathways in pancreatic cancer cells [48] or reduced the transformation phenotype by blocking c-Src and c-Met tyrosine kinases in osteosarcoma [49]. Whether the downregulation of Cav1 in tumor cells is correlated with changes in the mechanical phenotypes will need to be confirmed in the future. However, the cytoskeletal architecture of fibroblast cells is different from that of epithelial cells. It is possible that molecules other than Cav1 are involved in the regulation of epithelial cell mechanics.

Recently, mechanobiology (mechanical behaviors of living organisms) has become an intriguing and important field in the biomedical sciences [50]. In this study, we used Ha-Ras^V12^-transformed fibroblasts to demonstrate the importance of stiffness sensing in maintaining the cell number homeostasis of a tissue and to clarify the role of Cav1 in regulating cell transformation and the mechanical phenotype. In summary, changes in the mechanical phenotype, i.e. cell softening and the loss of stiffness sensing, desensitize cancer cells to soft matrix-induced growth arrest. This paper provides novel insights related to cancer mechanobiology that may be used to distinguish cancer cells from normal cells. Furthermore, the biomechanical characteristics of cells may serve as biomarkers to evaluate the state of transformation.

## MATERIALS AND METHODS

### Cell lines and culture conditions

Cell lines are detailed in the Supplementary materials and methods. Primary culture of cervical epithelia and HPDE cells were maintained in keratinocyte medium with the addition of Human Keratinocyte Growth Supplement (Invitrogen, Carlsbad, CA). BxPC-3, PANC-1, and AsPC-1 cells were maintained in RPMI medium 1640 (Invitrogen) supplemented with 10% fetal bovine serum (Invitrogen), penicillin (Sigma-Aldrich, St. Louis, MO), and streptomycin (Sigma-Aldrich). The other cell lines were maintained in Dulbecco's modified Eagle's medium (DMEM, Sigma-Aldrich) supplemented with 5% calf serum (Hyclone, Logan, UT), 2 mM L-glutamine (Invitrogen), penicillin, and streptomycin. All the cell lines were cultured at 37 °C in a 5% CO_2_, humidified incubator.

### Human samples

Informed consent was obtained from all subjects. The study was approved by the ethical committee of NCKU. Human cervical cancerous tissues were obtained from the operation room at NCKU Hospital. Once the tissue was harvested, the normal and cancerous areas were divided by an experienced surgeon; the normal cervix is characterized by its smooth and firm surface, while the cancerous area shows prominent protrusions identifiable to our naked eyes. The specimens were placed in ice-cold saline on a glass slide to keep them moist and transported to the AFM operation laboratory immediately. The moist samples were placed on the operation stage in AFM for measuring tissue stiffness. The preparation and measurement were completed within 20 min to reduce protease activity that may degrade tissue integrity.

### Establishment of IPTG-inducible Ha-Ras^V12^ expression in MDCK cells

The pSV*lac*O*Ras* and pHβlac*I*NLS*neo* plasmids were kindly provided by Dr. HS Liu [51] and were cotransfected into MDCK cells by the method of lipofection according to the manufacturer's instruction (Invitrogen). After antibiotic selection, G418 resistant cells were cloned and checked for Ras expression under IPTG induction. Colonies with inducible Ras protein or mRNA expression were picked and expanded in the absence of IPTG for further analysis.

### Inhibitors and plasmids

U0126 (MEK inhibitor) and PD 98059 (MEK inhibitor) were purchased from Calbiochem (Nottingham, UK) and dissolved in DMSO. Farnesylthiosalicylic acid (FTA, Cayman Chemical, Ann Arbor, Michigan) was purchased from Biomol (Plymouth Meeting, PA) and dissolved in DMSO. The Caveolin-1-Myc-mRFP plasmid was kindly provided by Dr. IR Nabi [52]. The RNA interference (RNAi) constructs shLacZ (TRCN0000072226), shCav1-1 (TRCN0000112662), and shCav1-2 (TRCN0000315312) were purchased from the National RNAi core facility, Institute of Molecular Biology/Genomic Research Center, Academia Sinica, Taipei, Taiwan.

### Fabrication of micropost arrays and quantification of traction force

Polydimethylsiloxane (PDMS) micropost arrays were fabricated using standard microfabrication techniques as previously described [14, 53] and detailed in the Supplementary materials and methods. Quantitative analysis of subcellular-level traction forces was performed as previously described [14, 53] and detailed in the Supplementary materials and methods.

### Immunofluorescence staining and confocal microscopy

Immunofluorescence staining was performed as previously described [34]. The primary antibodies used in this study were listed as follows: Cav1, β-catenin and E-cadherin (BD Biosciences Pharmingen; San Jose, CA), claudin-1 and ZO-1 (Invitrogen). After washing with PBS, the cells were incubated with the secondary antibody for anti-mouse or -rabbit IgG conjugated with Alexa 488 (Invitrogen-Molecular Probes) and/or phalloidin-TRITC (Sigma-Aldrich) and 10 μg/ml Hoechst 33258 (Sigma-Aldrich) for 1 h. The imaging was performed from sequential z-series scans with a FluoView™ *FV1000* confocal microscope (Olympus, Tokyo, Japan) with a 60 x water immersion lens, NA 1.35 (Uplsapo). Actin filaments in the apical, middle, and basal regions of a cell were recolored green, red, and blue, respectively. A Max XY projection image was generated from a stack of recolored confocal images using the ImageJ software (NIH).

### Western blot analyses

Western blot analysis was performed as previously described [34]. The cell lysates were harvested, resolved on SDS-PAGE, and then electrophoretically blotted onto nitrocellular paper. The blots were blocked with 5% nonfat dry milk in TBS-T and immunoblotted with specific primary antibodies, and then detected using horseradish peroxidase-conjugated secondary antibodies and made visible by fluorography with an enhanced chemiluminescence detection kit (GE Healthcare Life Sciences, Buckinghamshire, UK). The antibodies used in this study were listed as follows: Cav-1, FAK, β-catenin, and E-cadherin from BD Biosciences PharMingen; Cav-1 and β-actin from Abcam (Cambridge, MA); ^pY397^FAK and claudin-1 from Invitrogen; pERK and ERK from Cell Signaling (Boston, MA); Pan-Ras from Calbiochem; and RhoA and α-tubulin from Santa Cruz Biotechnology, Inc. (Santa Cruz, CA).

### Transwell migration assay

The migration ability was evaluated via a 24-well Transwell (8 μm pore size polycarbonate membrane, Corning). In brief, 5 × 10^4^ cells of cell clones were suspended in 300 μl of serum-free DMEM and seeded to the upper chamber, whereas 600 μl of DMEM containing 10% FBS and 10 μg/ml of collagen I was added to the outer side of the chamber. After being cultured in a 37° C, 5% CO_2_, humidified incubator for 6 h, cells on the upper surface of the membrane were removed by cotton-tipped swabs, and the penetrated cells on the lower membrane surface were fixed by 4% paraformaldehyde and stained with crystal violet. Cell migration values were determined by counting all penetrated cells of each clone under a phase contrast microscope (200 x magnitude) and then normalized with compared control as relative ratio. Some migration assays were evaluated using QCM™ Chemotaxis 8 μm and 3 μm 96-Well Cell Migration Assay kits (Chemicon, ECM 510 and ECM 515) according to the manufacturer's instructions and as detailed in the Supplementary materials and methods.

### Anchorage-independent growth assay

The anchorage-independent growth ability of various cell clones was determined by assessing colony formation efficiency in the soft agar system. Briefly, 1×10^4^ cells of each clone were suspended in 1.5 ml of 10% FBS-DMEM containing 0.3% low-melting agarose (Seakem LE). The suspension was then applied onto a layer of 10% FBS-DMEM containing 0.5% low-melting agarose in a well of six-well dish. After solidification, 2 ml of DMEM containing 10% FBS was then added and refreshed every three days. After 14–21 days, the colony numbers of each clone from three independent experiments were counted, and then normalized with the compared control as relative ratio.

### Evaluation of cell proliferation with Click-iT^®^EdU

Cell proliferation was evaluated by a Click-iT EdU Alexa Fluor 488 Imaging Kit (Invitrogen-Molecular Probes) as previously described [34] and as detailed in the Supplementary materials and methods.

### Measurements of cell/tissue mechanical properties by atomic force microscopy

For measurements of cell/tissue stiffness, a JPK NanoWizard^®^ II AFM with BioCell (JPK Instruments, Berlin, Germany) was equipped and manipulated as previously described [54]. Cells were trypsinized and replated on collagen- or fibronectin-coated dishes or coverslips at a density of 3–5 × 10^3^ cells/cm^2^ for 24 h. Before measurement, the media were changed to CO_2_-independent medium (Invitrogen). To investigate the E_eff_ of cells, tipless cantilevers (Arrow-TL1-50, Nanoworld, Neuchâtel, Swiss) modified with 5 μm (in diameter) polystyrene bead were used. The spring constants of all cantilevers were calibrated via thermal noise method in an experimental environment prior to each measurement and ranged from 0.02 to 0.08 N/m. The indenting force was set at 1 nN. The approaching and retracting rates of the cantilever were set at 1 μm/sec. The vertical working range of the cantilever piezo can be up to 15 μm. Force-distance curves were collected from the central top of the cell nucleus and calculated with the JPK package software, which was based on the Hertz model. For each cell line, a minimum of 60 cells were analyzed in two to three independent experiments. The data were presented as Mean ± SEM.

For measuring tissue stiffness, tipless cantilevers (CSC12-F, MikroMasch, Wetzlar, Germany) modified with 25 μm (in diameter) polystyrene bead were used. The spring constants of all cantilevers were calibrated via thermal noise method in liquid prior to each measurement and were valued at 0.08 N/m. The indenting force was set at 10 nN. The approaching and retracting rates of cantilever were set at 1 μm/sec. Force-distance curves were collected and calculated with the JPK package software, which was based on the Hertz model. The data were presented as mean ± SEM.

### Preparation and functionalization of polyacrylamide (PA) gel

PA gels with uniform stiffness were prepared according to a protocol by Chen et al. [34] and as detailed in the Supplementary materials and methods. PA gels from each polymerization batch were checked to verify consistent matrix mechanical properties by AFM. The Young's moduli of PA gels utilized in this study range as follows: Soft gel (S) represents 0.25 ± 0.06 kPa for 3% acrylamide, 0.08% bisacrylamide; and hard gel (H) represents 21.49 ± 1.24 kPa for 7.2% acrylamide, 0.5% bisacrylamide. PA gels with gradient stiffness were prepared according to the protocol of Tse and Engler [55] with minor modifications. Photomasks with a radial decrease in grayscale from 75% to 0% were designed in Photoshop (Adobe). Masks were printed on transparency sheets using a 600 dpi printer. A solution containing 8% acrylamide, 0.2% bis-acrylamide and 0.5% Irgacure 2959 (Ciba Specialty Chemicals, Tarrytown, NY) was prepared. Ten μL of polymer solution was sandwiched between a glass slide activated with SIGMACOTE^®^ and a 12 mm round glass coverslip activated with 3-aminopropyltrimethoxysilane. Then, the sandwiched solution was aligned on top of the photomask fixed on the surface of a benchtop UV transilluminator (UV2020-B, TopBio Co., Taiwan). The polymerization was initiated by exposure to 254 nm light for 2.5 min. After rinsing with PBS to remove unreacted monomer, PA gels were modified covalently with fibronectin (FN) with the photoactivatable sulfo-SANPAH (Pierce Biotechnology, Rockford, IL), followed by incubation with 10 μg/mL FN overnight at 4 °C, as previously described [6]. Finally, the gradient PA gels were rinsed well with PBS and soaked in the culture medium before plating the cells. The elastic modulus with respect to the distance from the center to the edge of the PA gel was measured by AFM. The uniformity of the functionalization was evaluated via immunofluorescence staining for FN by confocal microscopy. Figure 4A shows the representative result for the spatial stiffness gradient of the PA gel. The stiffness gradient spans from 0.2 to 30 kPa with a gradient strength of 7 ± 1.23 Pa/μm. Figure 4B shows that FN linked on the top surface of the PA gel with a roughly uniform distribution regardless of spatial changes in stiffness. To evaluate the spatial distribution of cells on the gradient gel, we performed crystal violet staining. Cells were fixed with 3.7% paraformaldehyde, and then stained with 0.05% crystal violet (*Sigma*). After being extensively washed with tap water, the samples were mounted in Vector mounting medium (H-1000) with the coverslip over it and photographed under a microscope for cell counts.

### Statistical analysis

Analyses of the results were performed using ANOVA and *t*-tests by GraphPad Prism 3.0 (GraphPad Software, San Diego, CA). Values of *p* < 0.05 were considered significant. Each experiment was repeated at least two or three times to ensure validity of the data. Most data are shown as the mean ± SEM of independent experiments. Some data were normalized as described in the figure legends and expressed as mean relative value ± SEM.

## SUPPLEMENTARY FIGURES


