# Supplementary material for: Mechanical phenotype of cancer cells: cell softening and loss of stiffness sensing
Source: Oncotarget. 2015 May 19;6(25):20946–58. doi: 10.18632/oncotarget.4173 (PMC4673241; doi:10.18632/oncotarget.4173)
Supplement: Supplementary file 1 [file oncotarget-06-20946-s001.pdf]

# Mechanical phenotype of cancer cells: cell softening and loss of stiffness sensing

## Supplementary Material

## Materials and Methods

### Cells lines

NIH3T3 cell and 7-4 cell [harboring the inducible Ha-Ras$^{V12}$ oncogene (pSVlacOras)] were kindly supplied by Dr. H.S. Liu (Department of Microbiology and Immunology, College of Medicine, National Cheng Kung University (NCKU), Taiwan). Human breast cell lines (M10, MCF7, MDA-MB 468) and human bladder cell lines (SVHUC-1, TSGH8301, and J82) were kindly supplied by Dr. M.D. Lai (Department of Biochemistry, College of Medicine, NCKU, Taiwan). NMuMG cell and MDCK cell were purchased from American Type Culture Collection (ATCC, Manassas, VA). Mouse breast cell lines (67NR and 4T1) were kindly supplied by Dr. C.Y. Chou (Department of Obstetrics & Gynecology, College of Medicine, NCKU, Taiwan). Human cervical cell lines (primary cervical epithelial, SiHa, HeLa) were kindly supplied by Dr. M.R. Shen (Department of Pharmacology, College of Medicine, NCKU, Taiwan). Human pancreatic cell lines (HPDE, BxPC-3, PANC-1, and AsPC-1) were kindly supplied by Dr. Y.S. Shen (Department of Surgery, NCKU Hospital, Taiwan).

### Preparation and functionalization of polyacrylamide (PA) gel

PA gel with uniform stiffness were prepared according to a protocol by Chen et al. (Chen et al., 2014). Briefly, solutions of varying acrylamide (Sigma-Aldrich) and bis-acrylamide (Sigma-Aldrich) concentrations were polymerized in the presence of ammonium persulfate

and tetramethylethylenediamine. Acrylic acid (0.3%, Sigma-Aldrich) was incorporated into the PA gel for further ECM crosslinking (Kandow et al., 2007). The gels were cast between a SIGMACOTE® (Sigma-Aldrich) activated glass coverslip and a 3-aminopropyltrimethoxysilane (Sigma-Aldrich) activated glass coverslip. After polymerization, the gels were washed extensively in 0.1 M MES (2-(N-morpholino) ethanesulfonic acid) (Sigma-Aldrich), pH 6. Subsequently, acrylic acid was activated by EDC (1-ethyl-3-(dimethylaminopropyl), 26 mg/ml in 0.1 M MES) (Pierce Biotech, Rockford, Illinois). After extensively washed with 0.1 M MES, 200 μg/ml type I collagen (BD Biosciences PharMingen, San Jose, CA) in 0.1 M MES was applied onto the PA gel and incubated overnight at 4 °C. Finally, the PA gels were rinsed well with PBS and soaked in the culture medium before plating the cells in an incubator under 5% $CO_2$ at 37 °C. PA gels from each polymerization batch were checked to verify consistent matrix mechanical properties by AFM.

**Fabrication of micropost arrays and quantification of traction force**

Polydimethylsiloxane (PDMS) micropost arrays were fabricated using standard microfabrication techniques as previously described (Fu et al., 2010; Yang et al., 2011). The fabricated silicon wafers were kindly gift from Dr. J Fu (Department of Mechanical Engineering, University of Michigan). In Brief, liquid PDMS prepolymer (Sylgard 184, Dow-Corning) was poured over a silicon master, cured at 110 °C for 15 min, and peeled to create a negative replica containing an array of holes. The negative replica were plasma-oxidized and silanized to facilitate subsequent release of PDMS from the replica. PDMS prepolymer was poured over the negative replica and cured at 110 °C for 20 h, after which the PDMS micropost arrays were peeled from the negative replica. To print FN on the tips

of micropost, PDMS Stamps were made by casting Sylgard 184 (Dow Corning, Midland, MI) liquid prepolymer, curing, washing with ethanol, and drying under nitrogen. Stamps were then immersed for 1 hour in an aqueous solution of FN, rinsed thoroughly with deionized water, blown dry under nitrogen and placed in conformal contact with plasma-treated micropost arrays. The micropost arrays were then blocked with Pluronic F127 (BASF), and used under standard culture conditions.

Quantitative analysis of subcellular level traction forces was performed as previously described (Fu et al., 2010; Yang et al., 2011). Briefly, the micromolded PDMS micropost arrays underlying cells were imaged with an Olympus FV-1000. For each cell, fluorescent images of the 9-DiI-stained PDMS microposts were acquired at two different focal planes. The top and bottom images of micropost arrays was acquired at the focal plane passing through the top and bottom surfaces of the microposts, respectively. These two images were analyzed with both Olympus FV-1000 software and custom-developed MatLab program to calculate traction forces.

**Transwell migration assay**

Some migration assay were evaluated via QCM™ Chemotaxis 8 µm and 3 µm 96-Well Cell Migration Assay kit (Chemicon, ECM 510 and ECM 515) according to the manufacturer’s instructions. In detail, 2 x $10^4$ cells suspended in serum free DMEM were seeded to the upper chamber, whereas DMEM containing 10% FBS was added to the outer side of the chamber. After being cultured for 6 h (8 µm pore) or 20 h (3 µm pore), penetrated cells on the lower membrane surface were released by cell detachment solution. These detached cells were subsequently lysed and detected by the patented CyQuant GR dye (Molecular Probes). Finally, the sample were read with a fluorescence plate reader

using 480/520 nm filter set and then normalized with the compared control as relative ratio.

**Evaluation of cell proliferation with Click-iT®EdU**

The cells were fed 10 μM EdU under regular growth conditions for 1 h prior to analysis. After fixed and permeabilized, incorporated EdU were visualized using the Click-iT ®EdU Alexa Fluor ®488 Imaging kit (green). Cells were counter stained with 1 μg/ml Hoechst 33342 (Blue). Images were acquired with a 20 X objective on a Nikon Eclipse 800 epifluorescence microscope. The DNA synthesis was quantifying the ratios of EdU-positive cell count to Hoechst 33342-positive cell count. The results were normalized to corresonding reference values, which are for cells cultured on G. Data are presented as mean relative value ± SEM.

**Supplementary Figures and Figure legend**

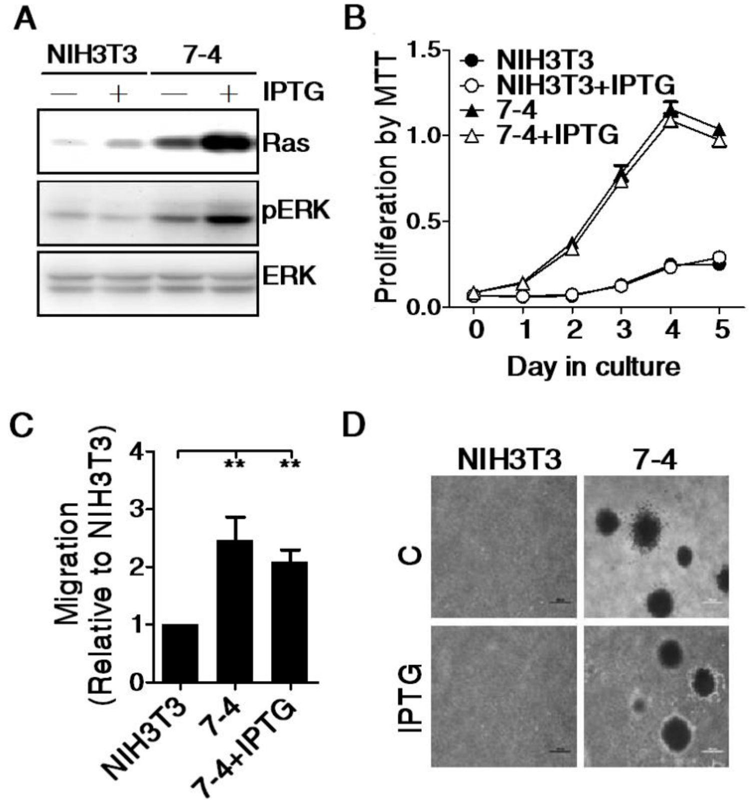


**Supplementary Figure 1: Oncogenic Ha-Ras$^{V12}$ induces transformation in 7-4 cells.** (A) Western blot analysis for the induction of Ha-Ras$^{V12}$ expression under IPTG treatment for 24 h in NIH3T3 and 7-4 cells. (B) Both 7-4 and IPTG-treated 7-4 cells showed much higher proliferation rates by MTT assay. (C) Both 7-4 and IPTG-treated 7-4 cells exhibited elevated migration as compared to NIH3T3 cells in the Transwell migration assay. (D) Both 7-4 cells and IPTG-treated 7-4 cells exhibited anchorage independent growth and formed colonies in soft agar assay. All data were expressed as the mean ± SEM from two or three experiments. * $P<0.05$, ** $P<0.01$, *** $P<0.001$. NS = not significant. Scale bar = 100 μm.

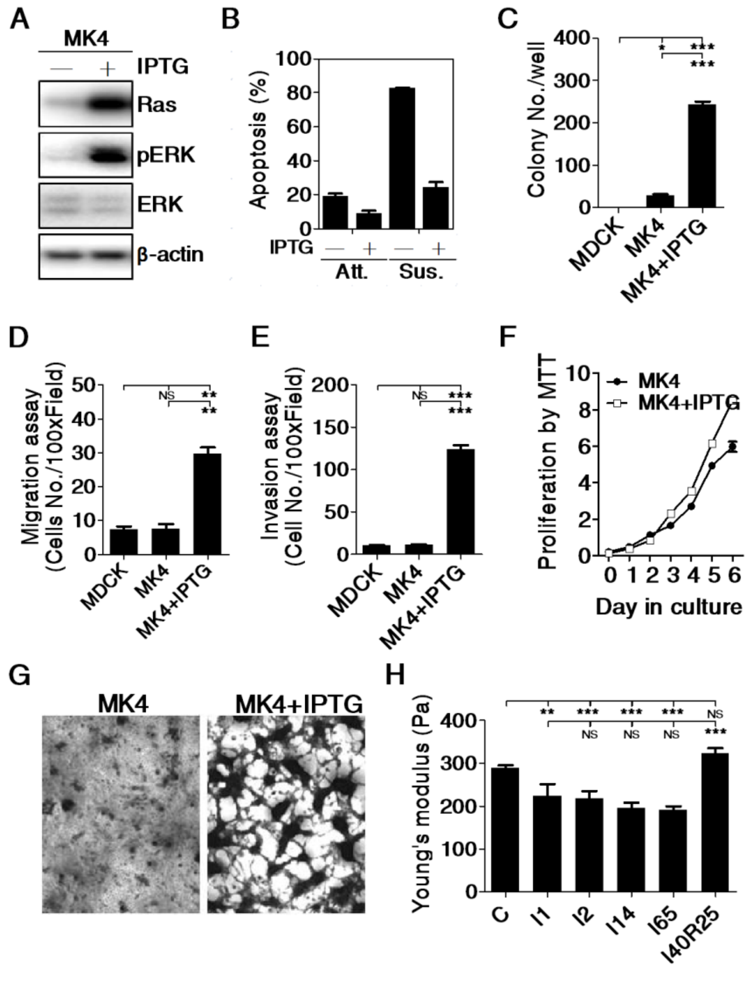


**Supplementary Figure 2: Oncogenic Ha-Ras$^{V12}$ induces transformation in MK4 cells.** (A) Western blot analysis for the induction of Ha-Ras$^{V12}$ expression under IPTG treatment for 24 h in MK4 cells. (B) Ha-Ras$^{V12}$ overexpressed MK4 cells were resistant to anoikis. (C) Ha-Ras$^{V12}$ overexpressed MK4 cells showed anchorage independent growth and formed colonies in soft agar assay. (D) Ha-Ras$^{V12}$ overexpressed MK4 cells exhibited elevated migration ability in Transwell migration assay. (E) Ha-Ras$^{V12}$ overexpressed MK4 cells exhibited higher invasion ability. (F) Ha-Ras$^{V12}$ overexpressed MK4 cells slightly enhanced proliferation rate by MTT assay. (G) Ha-Ras$^{V12}$ overexpressed MK4 cells tended to form foci after long term culture. (H) Ha-Ras$^{V12}$ induced-cell softening was reversible after withdrawal of IPTG. All data were expressed as the mean ± SEM. *p< 0.05, ** P<0.01, *** P<0.001, NS = not significant.

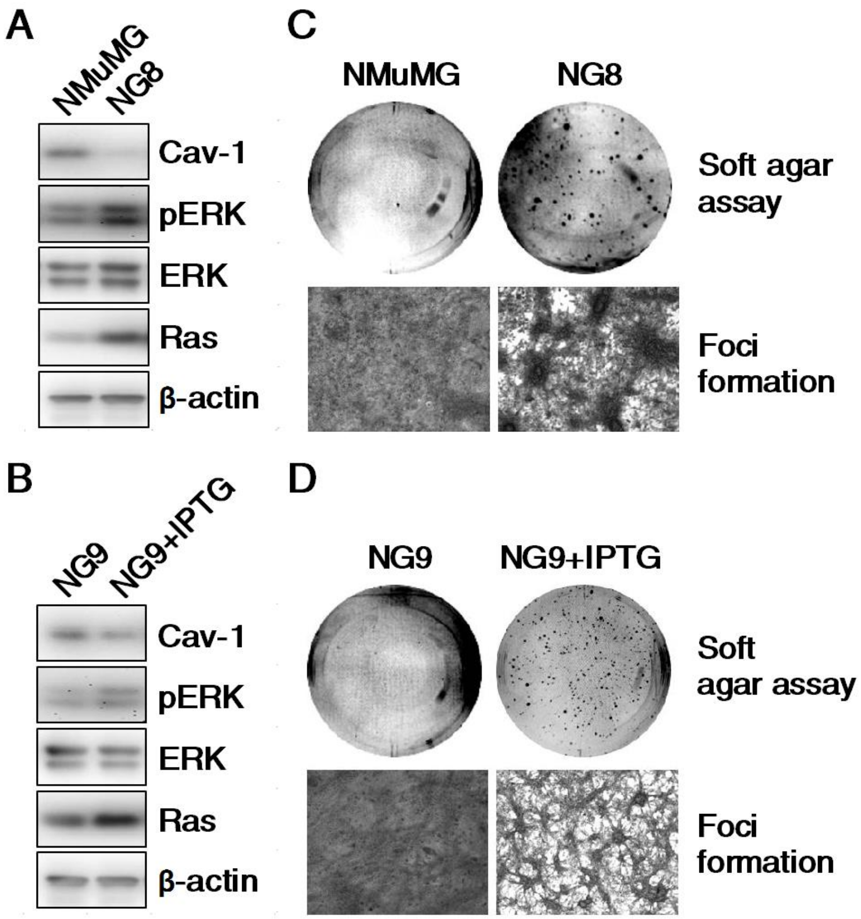


**Supplementary Figure 3: Oncogenic Ha-Ras$^{V12}$ induces transformation in NMuMG cells.** (A and B) Western blot analysis for the Ha-Ras$^{V12}$ expression in NMuMG, NG8 and NG9 treated with or without IPTG. Both (C) Ha-Ras$^{V12}$ constitutively expressed NG8 cells and (D) IPTG-induced Ha-Ras$^{V12}$ expressed NG9 cells showed anchorage independent growth in soft agar assay, and tended to form foci after long term culture.

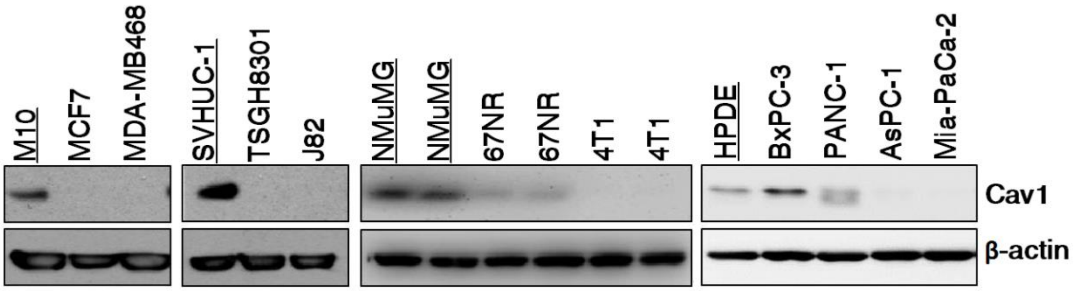


**Supplementary Figure 4: Cav1 levels are downregulated in most cancer cell lines.** Normal cell lines (underlined) and cancer cell lines were cultured on tissue culture dishes overnight and then harvested for Western blot analysis. β-actin served as a loading control.

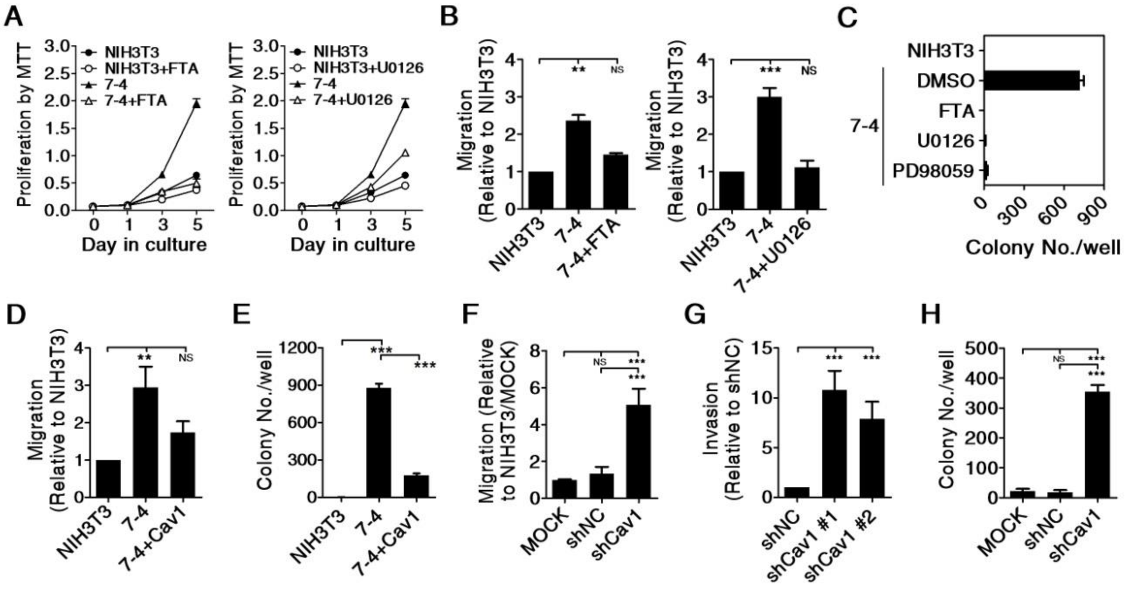


**Supplementary Figure 5: The expression levels of Cav1 is negatively correlated with cell transformation in fibroblast NIH3T3 cells.** Ras inhibitor (FTA) and MEK1/2 inhibitor (U0126) suppressed Ha-Ras$^{V12}$-induced increase in (A) proliferation, (B) migration, (C) anchorage independent cell growth in 7-4 cells. Overexpression of Cav1 in 7-4 cells suppressed Ha-Ras$^{V12}$-induced increase in (D) migration, (E) anchorage independent cell growth. Knockdown of Cav1 in NIH3T3 cells caused increase in (F) migration, (G) invasion, and (H) anchorage independent cell growth. All data were expressed as the mean ± SEM from two or three experiments. *p< 0.05, ** P<0.01, *** P<0.001, NS = not significant.

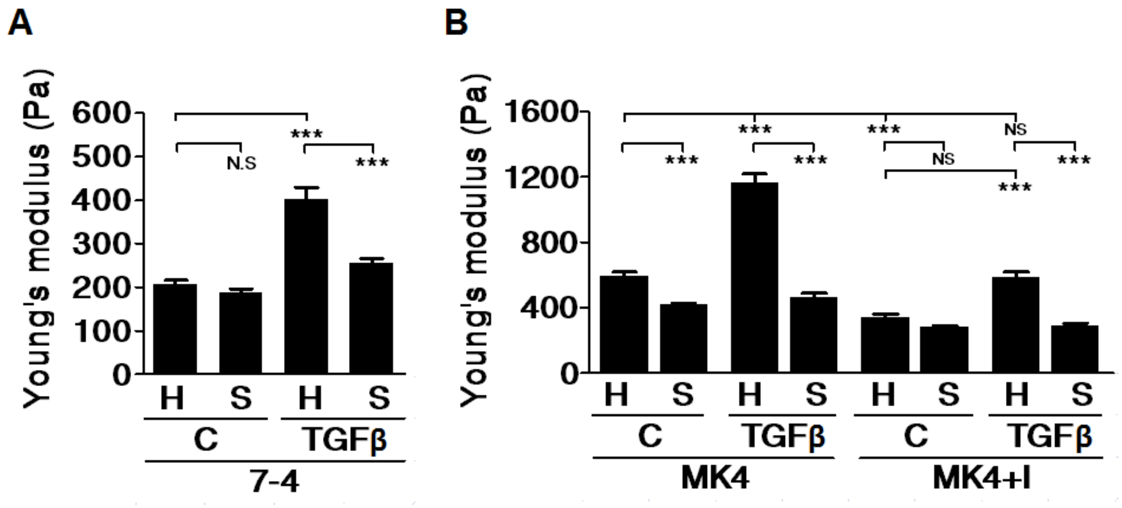


**Supplementary Figure 6: TGFβ treatment not only increases cell stiffness but also rescues rigidity sensing in Ha-Ras$^{V12}$ transformed cells.** Cells were plated on cultured dish and treated with or without TGFβ (10 ng/ml) for 24 h. Then, the cells were replated on hard PA gel (H, E≒20 kPa) or soft PA gel (S, E≒0.2 kPa) and treated with TGFβ (10 ng/ml) for another 24 h. The effective Young's moduli of cells were measured by Bio-AFM. (A) 7-4 cells, (B) MK4 and MK4+I cells. All data were expressed as the mean ± SEM. *** P<0.01, NS=not significant.

## References:

Chen, W.C., Lin, H.H., and Tang, M.J. (2014). Regulation of proximal tubular cell differentiation and proliferation in primary culture by matrix stiffness and ECM components. American journal of physiology. Renal physiology *307*, F695-707.

Fu, J., Wang, Y.K., Yang, M.T., et al. (2010). Mechanical regulation of cell function with geometrically modulated elastomeric substrates. Nat Methods *7*, 733-736.

Kandow, C.E., Georges, P.C., Janmey, P.A., et al. (2007). Polyacrylamide hydrogels for cell mechanics: steps toward optimization and alternative uses. Methods Cell Biol *83*, 29-46.

Yang, M.T., Reich, D.H., and Chen, C.S. (2011). Measurement and analysis of traction force dynamics in response to vasoactive agonists. Integr Biol (Camb) *3*, 663-674.
